# Functional Relationship Between T15 and J558 Idiotypes in BALB/c Mice

**DOI:** 10.1155/1991/91729

**Published:** 1991

**Authors:** Meenal Vakil, John F. Kearney

**Affiliations:** 1 Division of Developmental and Clinical Immunology Department of Microbiology, and the Comprehensive Cancer Center University of Alabama at Birmingham Birmingham Alabama 35294 USA; 2 263 Tumor Institute University of Alabama at Birmingham UAB Station Birmingham Alabama 35294 USA

## Abstract

In inbred strains of mice, antiphosphorylcholine (PC) and anti-α1,3 dextran (DEX). antibodies are structurally distinct from each other and have been shown to exhibit noncrossreactive antigen binding and idiotypic specificities. However, the prototype anti-PC and anti-DEX antibodies, TEPC15 and J558, respectively, were shown to be connected via a common autoantiidiotypic monoclonal antibody isolated from newborn BALB/c mice. The capacity of various monoclonal anti-PC and anti-DEX antibodies as well as the antigens PC and DEX to modulate T15 and J558 idiotypes in BALB/c mice was tested by their administration to newborn mice. Anti-PC antibodies of the .T15 idiotype injected into 2-4-day-old mice, at a time when T15 anti-PC precursors develop in BALB/c mice, suppressed the anti- PC response of these mice at 6 weeks of age. Similarly, J558 antibodies injected into 8-12-day-old mice, at a time when J558 precursors normally develop, suppressed the response to DEX. As a further demonstration of this connectivity, the injection of J558 into 4-day-old mice led to a down modulation of T15 idiotype, whereas both T15 and a minor idiotypeexpressing antibody M167 when injected into 8-12-day-old mice caused a reduction in expression of the J558 idiotype. As predicted from in vitro analysis, injection of anti-PC antibodies of the M167 idiotype 2 to 4 days after birth enhanced the subsequent response to PC. However, anti-PC antibodies expressing another minor M603 idiotype did not affect the PC. response. The results parallel the *in vitro* enhancement of M167 antibodies but not M603 on T15 binding to antiidiotype *in vitro*. Similarly, anti-DEX antibodies expressing the M104E idiotype had no detectable effects on the capacity to respond to PC or DEX or on the expression of T15 and J558 idiotypes as adults. Exposure of newborn mice to PC led to a dramatic reduction in the response to DEX as adults, whereas exposure to DEX at this stage of development had no effect on response to PC as adults. Collectively, these observations provide evidence for a complex functional connectivity between T15 and J558 idiotype-bearing B cells during ontogeny and extend our previous observations that development of these idiotypes is regulated by idiotype-directed interactions between B cells or their immunoglobulin products.


*Developmental Immunology*, 1991, Vol. 1, pp. 213–224
Reprints available directly from the publisher
Photocopying permitted by license only

© 1991 Harwood Academic Publishers GmbH
Printed in the United Kingdom

### Functional Relationship Between T15 and J558 Idiotypes in BALB/c Mice

MEENAL VAKIL*† and JOHN F. KEARNEY†

†*Division of Developmental and Clinical Immunology, Department of Microbiology, and the Comprehensive Cancer Center, University of Alabama at Birmingham, Birmingham, Alabama 35294*

In inbred strains of mice, antiphosphorylcholine (PC) and anti-α1,3 dextran (DEX) antibodies are structurally distinct from each other and have been shown to exhibit noncross-reactive antigen binding and idiotypic specificities. However, the prototype anti-PC and anti-DEX antibodies, TEPC15 and J558, respectively, were shown to be connected via a common autoantiidiotypic monoclonal antibody isolated from newborn BALB/c mice. The capacity of various monoclonal anti-PC and anti-DEX antibodies as well as the antigens PC and DEX to modulate T15 and J558 idiotypes in BALB/c mice was tested by their administration to newborn mice. Anti-PC antibodies of the T15 idiotype injected into 2–4-day-old mice, at a time when T15$^+$ anti-PC precursors develop in BALB/c mice, suppressed the anti-PC response of these mice at 6 weeks of age. Similarly, J558 antibodies injected into 8–12-day-old mice, at a time when J558 precursors normally develop, suppressed the response to DEX. As a further demonstration of this connectivity, the injection of J558 into 4-day-old mice led to a down modulation of T15 idiotype, whereas both T15 and a minor idiotype-expressing antibody M167 when injected into 8–12-day-old mice caused a reduction in expression of the J558 idiotype. As predicted from *in vitro* analysis, injection of anti-PC antibodies of the M167 idiotype 2 to 4 days after birth enhanced the subsequent response to PC. However, anti-PC antibodies expressing another minor M603 idiotype did not affect the PC response. The results parallel the *in vitro* enhancement of M167 antibodies but not M603 on T15 binding to antiidiotype *in vitro*. Similarly, anti-DEX antibodies expressing the M104E idiotype had no detectable effects on the capacity to respond to PC or DEX or on the expression of T15 and J558 idiotypes as adults. Exposure of newborn mice to PC led to a dramatic reduction in the response to DEX as adults, whereas exposure to DEX at this stage of development had no effect on response to PC as adults. Collectively, these observations provide evidence for a complex functional connectivity between T15 and J558 idiotype-bearing B cells during ontogeny and extend our previous observations that development of these idiotypes is regulated by idiotype-directed interactions between B cells or their immunoglobulin products.

KEYWORDS: B-cell development, dominant idiotypes, autoantiidiotype, connectivity

### INTRODUCTION

In mammals, antibody responses to certain defined antigens including bacterial polysaccharides are restricted to the expansion of one or a few clones of B cells resulting in idiotypic dominance of antibodies produced by these clones in the serum (Blomberg et al., 1972; Claflin et al., 1974; Pawlak and Nisonoff, 1973; Lieberman et al., 1976; Hansberg et al., 1977; Mäkelä and Karjalainen, 1977; Capra et al., 1977). It was shown that production of antibodies expressing these dominant idiotypes could be modulated by passive transfer of antiidiotypic antibodies (Cosenza and Köhler, 1972; Pawlak et al., 1973; Eichman, 1974). In addition to the numerous demonstrations of direct effects of polyclonal as well as monoclonal antiidiotypic antibodies on antibody responses *in vivo*, evidence has also accumulated for the existence of regulatory B-cell networks (Cosenza and Köhler,

*Corresponding author. Present address: 263 Tumor Institute, University of Alabama at Birmingham, UAB Station, Birmingham, Alabama 35294.

1972; Cazenave, 1977; Kelsoe and Cerny, 1979; Bona et al., 1981; Rajewsky and Takemori, 1983; Pollok and Kearney, 1984).

During attempts to determine whether such idiotype-directed interactions occur under physiologically relevant situations, we isolated B-cell hybridoma-derived antibodies from perinatal mice that react with structurally and genetically defined immunoglobulin idiotypes (Vakil and Kearney, 1986; Kearney et al., 1987). In addition, extensive connectivity, which is idiotype-associated, has been demonstrated between these perinatally derived antibodies (Holmberg et al., 1984; Kearney et al., 1987). Based on the binding characteristics of these monoclonal antibodies *in vitro* and the demonstration that administration of these antibodies has potent long-lived effects on the expression of target idiotypes *in vivo* (Vakil and Kearney, 1986), we proposed that B cells interact through immunoglobulin idiotypic determinants and that these interactions are involved in the establishment of the adult B-cell repertoire.

A monoclonal autoantiidiotypic antibody BD2 was shown to regulate the capacity of BALB/c mice to respond to both phosphorylcholine (PC) and $\alpha$1,3 dextran (DEX). Furthermore, the nature and extent of these modulating effects depended on the age at which BD2 was injected into mice. Additionally, we also demonstrated that inactivation of similar early appearing antiidiotypic B cells *in vivo* led to a lack of development of T15- and J558-like B cells and a diminished capacity to respond to PC and DEX (Vakil et al., 1986). Based on these criteria, we proposed that idiotypic interactions among complementary sets of B cells during ontogeny were required for the development of T15 and J558 idiotypes.

In this report, we have extended these studies by analyzing the effects of administration of soluble idiotype-bearing antibodies and antigen on the development of these germline-encoded responses to PC and DEX. We demonstrate that development of the dominant idiotypes T15 and J558 can be altered by neonatal exposure to the antibodies expressing either T15 or J558 idiotypes as well as antigens PC and DEX. These observations establish a functional relationship between idiotypically connected B cells and suggest that such idiotypic connections may regulate the program of repertoire development.

### RESULTS

### Characteristics and Reactivity Patterns of Idiotype-Bearing Antibodies

Antigen-responsive T15-like and J558-like B cells in BALB/c mice first appear between 5 to 7 days and 12 to 15 days after birth, respectively (Sigal et al., 1977; Stohrer and Kearney, 1984). Since our hypothesis proposes that idiotypic interactions at a receptor level are essential for the selective expansion of $T15^+$ and $J558^+$ B cells, we wished to test whether passive administration of soluble idiotype-bearing antibodies during this period would interfere with the normal development of T15- and J558-like B cells. Table 1 summarizes the antigen-binding and idiotypic characteristics of the antibodies used in these studies.

TABLE 1
Characteristics of Antibodies Used for Neonatal Treatments

| Ab name | Isotype | Reactivity | Idiotype sequences | $V_H$ and $V_L$ |
|---|---|---|---|---|
| TEPC15[a] | IgA,$\kappa$ | PC | T15 | Germline |
| BH8[b] | IgM,$\kappa$ | PC | T15 | Germline |
| M167[c] | IgA,$\kappa$ | PC | M167/M511 | Mutated |
| HPCM27[d] | IgM,$\kappa$ | PC | M167/M511 | Germline |
| HCM25[d] | IgM,$\kappa$ | PC | M603 | Germline |
| J558[e] | IgA,$\lambda$1 | DEX | J558 | Germline |
| M104E[f] | IgM,$\lambda$1 | DEX | M104E | Germline |
| 4F-1[g] | IgM,$\kappa$ | M167 | —[h] | —[h] |

[a]Gearhart et al. (1981).

[b]Pollok et al. (1984).

[c]Rudikoff and Potter (1976).

[d]Perlmutter et al. (1984).

[e]Schilling et al. (1980).

[f]Kehry et al. (1979).

[g]Kang and Köhler (1990).

Shown in Fig. 1A is a diagrammatic summary of the reactivities of several of these well-characterized monoclonal antibodies that bind to either PC or DEX. Also included are the monoclonal antiidiotypic antibodies BD2, DB3, and 4F-1. BD2 derived from 2-day-old mice is an autoantiidiotypic antibody that reacts with T15, M167, and J558 (Fig. 1B). DB3, derived from fetal mice, in turn, reacts with BD2 (Vakil and Kearney, 1986). 4F-1 was raised by immunization of adult BALB/c mice with a preparation of purified M167 and recognizes a determinant unique to M167 not expressed by T15 or J558 (Kang and Köhler, 1990). We have previously demonstrated that injection of BD2 and DB3 into newborn mice can enhance the adult antibody responses to PC and DEX with T15 and J558 as major idiotypes. In the present study, each of the antibodies shown in Fig. 1, except BD2 and DB3, was injected into neonatal BALB/c mice at various times after birth coincident with the normal appearance of T15- and J558-like B cells during ontogeny. All animals treated neonatally with these antibodies were then challenged when 6 to 7 weeks old with *S. pneumoniae* strain R36A, or DEX, and their anti-PC and anti-DEX responses analyzed to assess the modulatory effects of the neonatal treatments.

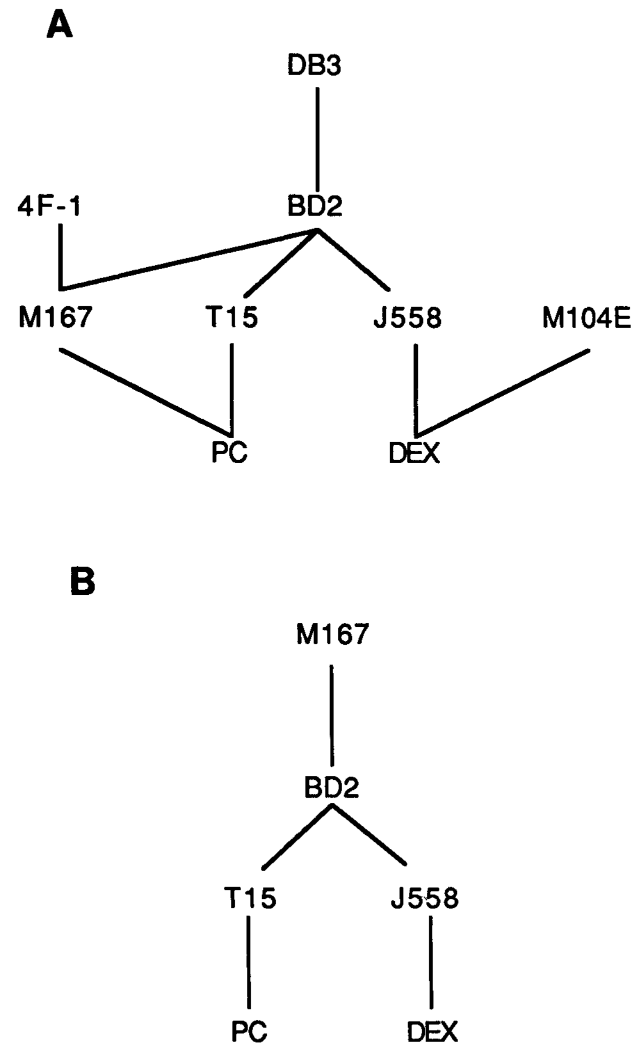

FIGURE 1. A network describing idiotypic relationships among antibodies used in these studies as ascertained by *in vitro* binding analysis and their relationship to the antigens PC and DEX. (A) See text for description. (B) See Discussion.

### Effects of Dominant Idiotypes T15 and J558 on the Antibody Response to PC

In mice injected with 10 μg of T15$^+$ IgA (TEPC15) or IgM (BH8) antibodies 2 to 4 days after birth, there was an approximate tenfold reduction of the antibody response to PC following challenge with R36A at the age of 6 weeks (Fig. 2A). Partial reduction of antibody levels expressing the T15 idiotype was induced when treatment was delayed until 8 days after birth (results not shown). In mice injected with the same antibodies on day 10, the response to PC was not significantly different as compared to that in saline-treated controls (Fig. 2B). Injection of 10 μg of the non-PC binding IgA antibody J558 on day 2 led to partial suppression of T15 idiotype but only 15 to 20% reduction in the total antibody response to PC. In contrast, the response to PC in mice treated with J558 10 days after birth was no different than in saline-treated control mice in magnitude or idiotype profile (Fig. 2B). To test the possibility that the injected antibody forms antigen-antibody complexes with PC that may be responsible for suppression of endogenous T15 idiotype-bearing B cells, U10, a non-PC-binding somatic variant of S107, an IgA T15$^+$ antibody (Behar et al., 1989) was injected into 2-day-old mice. This treatment also resulted in a reduction of the response to PC (Fig. 2A) suggesting that the T15 idiotypic determinants on the soluble mutant non-PC-binding molecule U10 rather than antigen were responsible for the block in development of endogenous T15$^+$ B cells.

### Effects of Dominant Idiotypes T15 and J558 on the Antibody Response to DEX

In mice that received 10 μg of J558$^+$ antibodies 2 to 4 days after birth, the total response to DEX and

J558 idiotype was reduced as compared to that in saline-treated controls (Fig. 3A). The anti-DEX response was also reduced by treatment with T15 antibodies between days 2 and 4. However, injection of J558 on day 10 resulted in a reduced response to DEX and completely abolished the production of the J558 idiotype (Fig. 3B). Partial reduction of the response to DEX and J558 idiotype was still evident in mice treated with 10 $\mu g$ of J558 antibody as late as 15 days after birth (results not shown). Treatment with T15 idiotype on day 10 also brought about a severe down modulation of the J558 idiotype (Fig. 3B).

These experiments showed that administration of exogenous soluble T15 or J558 into neonatal mice resulted in the suppression of these idiotypes when they were challenged as adults with the appropriate antigen. Furthermore, this idiotypic suppression was induced most efficiently at critical periods during development. In accord with our previous studies, these periods coincided with the developmental windows when B-cell clones expressing T15 or J558 idiotypes are normally generated in BALB/c mice (Stohrer and Kearney, 1984; Vakil et al., 1991).

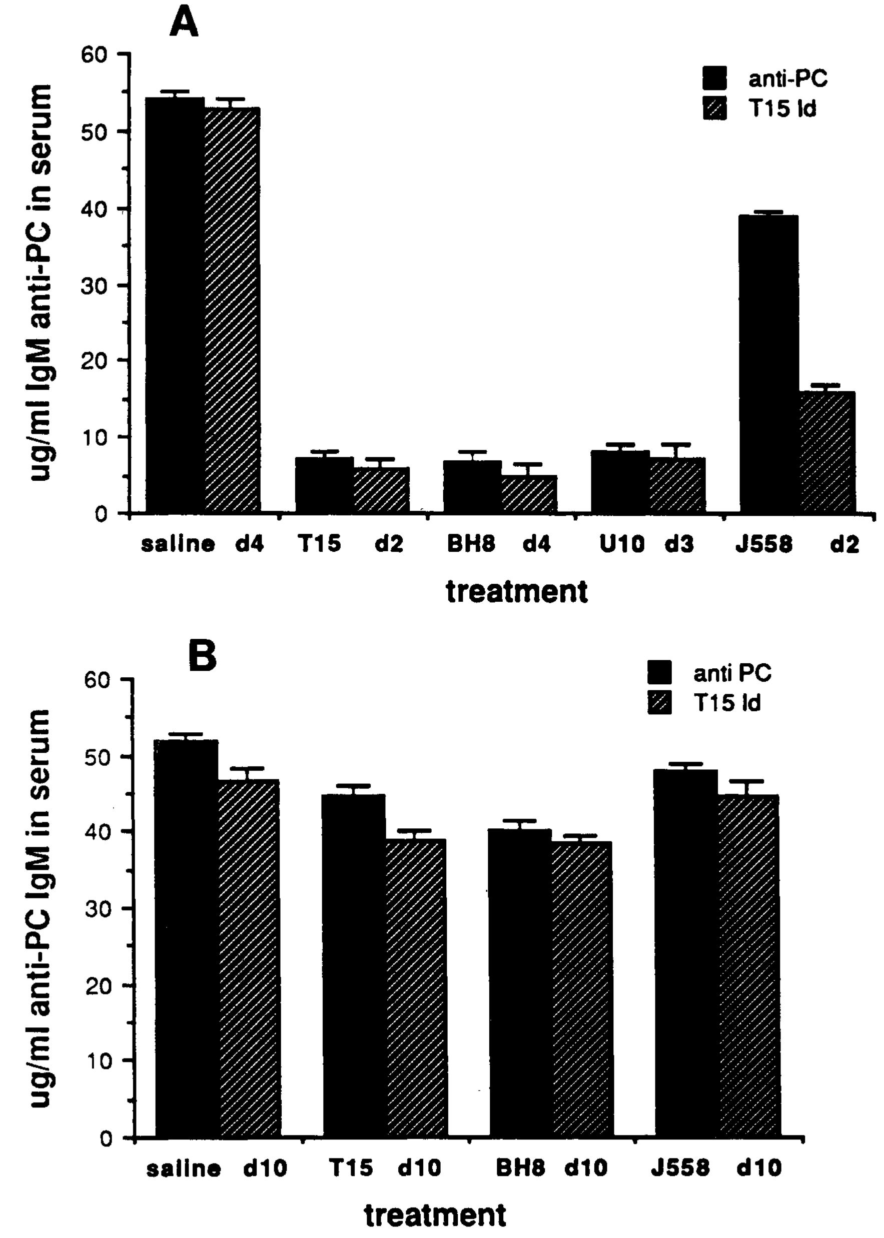

FIGURE 2. Effects of neonatal administration of T15 and J558 idiotype-bearing antibodies on the subsequent antibody response to PC. Levels of anti-PC antibodies (solid bars) and T15$^{+}$ antibodies (cross-hatched bars) in saline-treated control mice and in antibody-treated mice. (A) Response in mice treated with antibodies immediately preceding the development of endogenous T15 B cells and challenged as adults with R36A. (B) Response in mice treated with antibodies immediately after the development of endogenous T15 B cells but preceding that of J558 B cells and challenged as adults with R36A.

### Effects of Minor Idiotypes M167, M603, and M104E on the Antibody Responses to PC and DEX

In addition to the normally dominant T15 idiotype, a small portion of the normal adult BALB/c response to PC consists of antibodies expressing the M167 or M603 idiotype (Claflin, 1976). Similarly, antibodies expressing the M104E idiotype represent a variable minor but significant portion of the response to DEX (Stohrer and Kearney, 1984). We have previously reported that a monoclonal antiidiotype antibody BD2 derived from 2-day-old BALB/c mice also reacts with antibodies expressing the M167 idiotype but not the M603 or M104E idiotypes (Vakil and Kearney, 1986). We, therefore, chose to investigate whether injection of antibodies expressing these idiotypes into neonatal mice would alter their capacity to respond to antigens PC and DEX. As

A

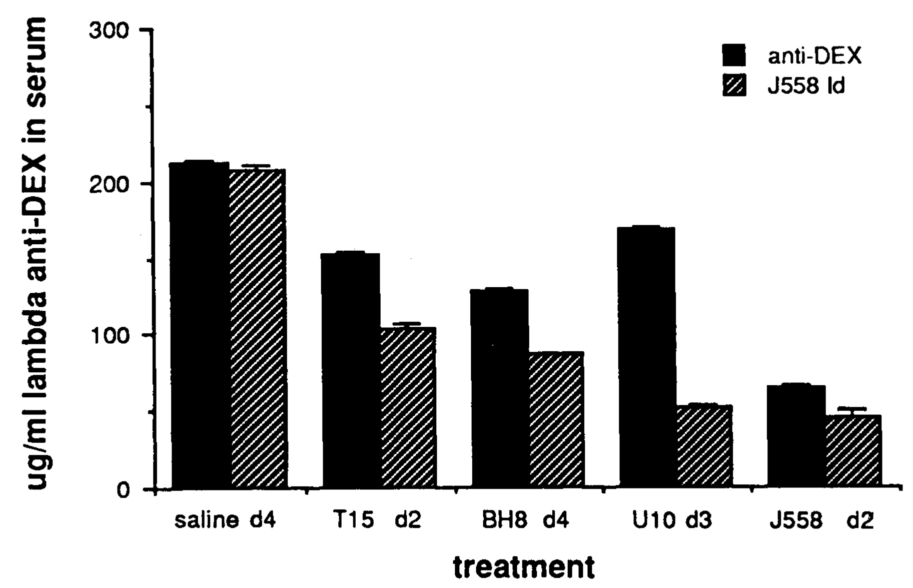

B

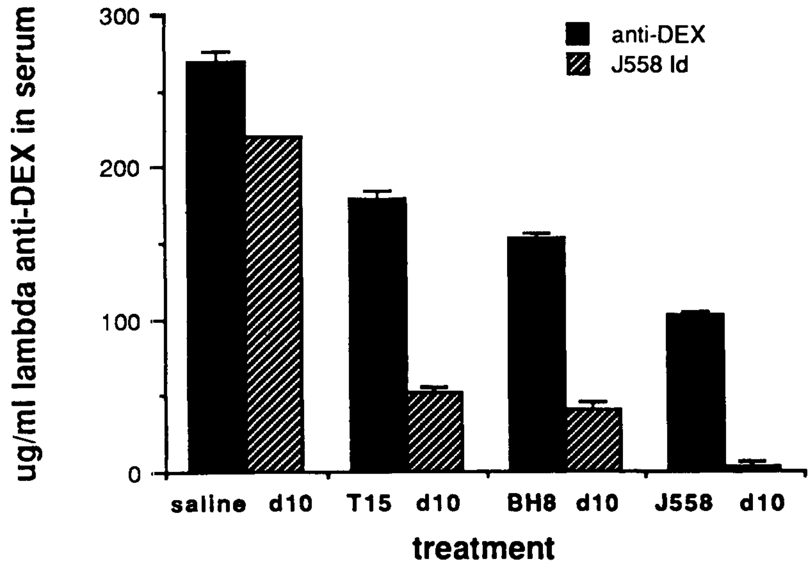

FIGURE 3. Effects of neonatal administration of T15 and J558 idiotype-bearing antibodies on the subsequent antibody response to DEX. Levels of anti-DEX antibodies (solid bars) and J558$^+$ antibodies (cross-hatched bars) in saline-treated control mice and in antibody-treated mice. (A) Response in mice treated preceding the development of endogenous T15 B cells and challenged as adults with dextran. (B) Response in mice treated after the development of endogenous T15 B cells but preceding that of J558 B cells and challenged as adults with dextran.

shown in Fig. 4A, injection of 10 $\mu g$ of the IgM antibody HPCM27, which expresses the M167 idiotype on day 4, led to a threefold increase in the total PC-specific IgM in the serum when these mice were challenged with R36A as adults, with T15 as the dominant idiotype. This treatment also led to a loss of J558 dominance (Fig. 4B) in response to DEX. However, treatment with HPCM27 on day 8 or 12 did not induce significant alteration of the antibody responses to PC (Fig. 4A), but led to a diminished response to DEX and reduction of J558 idiotype (Fig. 4B). Similar results were obtained when the IgA

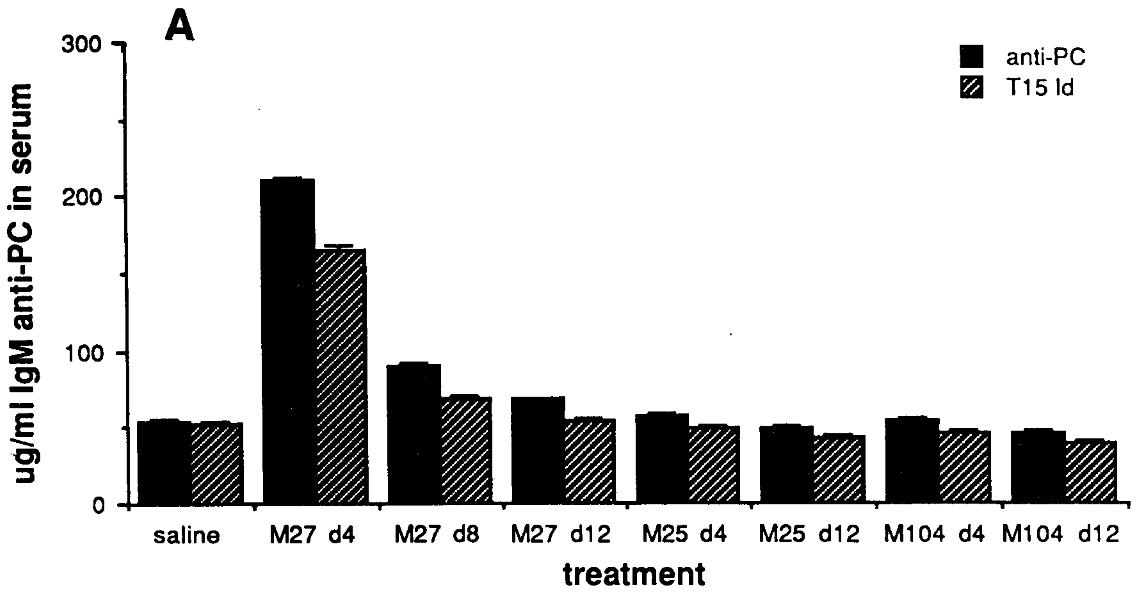

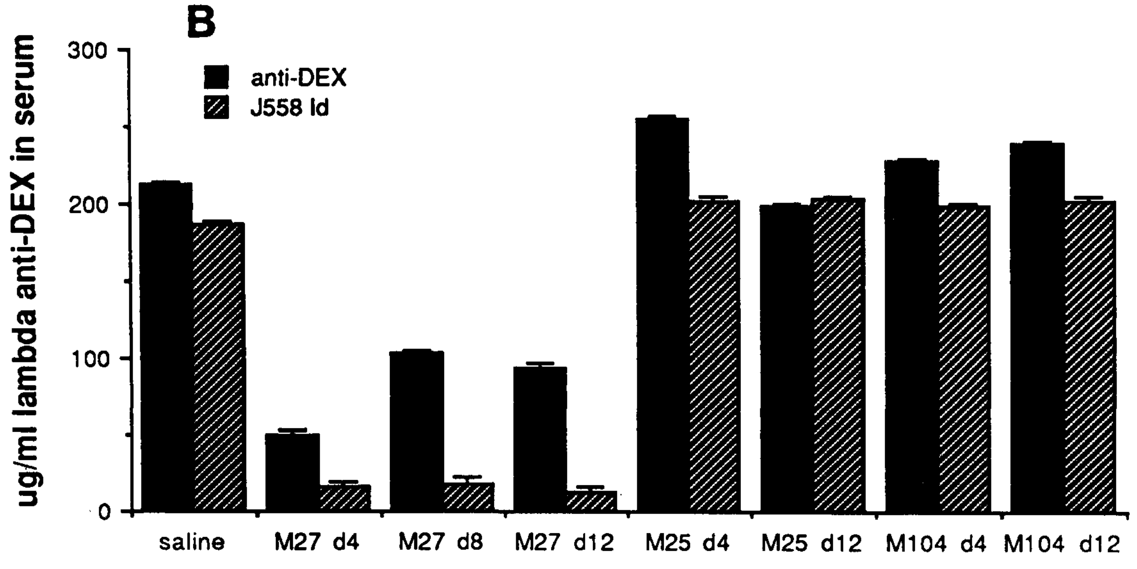

FIGURE 4. Effects of neonatal administration of HPCM27, HPCM25, and M104E antibodies (see Table 1 for description) on the subsequent antibody responses to PC and DEX. (A) Levels of anti-PC antibodies (solid bars) and $T15^+$ antibodies (cross-hatched bars) in control mice and in mice treated with antibodies preceding or after the development of endogenous T15 B cells and challenged as adults with R36A. (B) Levels of anti-DEX antibodies (solid bars) and $J558^+$ antibodies (cross-hatched bars) in control mice and in mice treated with antibodies preceding or after the development of endogenous T15 B cells and challenged as adults with dextran.

antibody M167 was injected into neonatal mice (results not shown). On the other hand, injection of HPCM25, an IgM antibody bearing the M603 idiotype, or M104E, an IgM anti-DEX antibody, on days 4 or 12 failed to modulate antibody responses to PC or DEX (Figs. 4A and 4B). Thus, M167 but not M603 or M104E injected into neonatal mice at critical times can modulate the antibody responses to PC and DEX, which suggests that B-cell clones expressing the minor idiotype M167 may be actively involved in the development of the dominant T15 idiotype.

In order to investigate the possibility that M167-like B cells play a role in the development of T15- and J558-like B cells in neonatal mice, the following experiment was conducted. Fifty $\mu g$ of 4F-1, a monoclonal antibody specifically reactive with M167 but not the T15 idiotype, was injected into neonatal mice on days 2 and 4 in an attempt to alter the functional development of endogenous B cells expressing the M167 idiotype. When mice treated in this fashion were challenged with R36A 6 to 8 weeks later, their antibody response to PC was only 30% of that in saline-treated control mice with almost total loss of T15 idiotype although 4F1 does not react with $T15^+$ antibodies (Fig. 5A). However, this

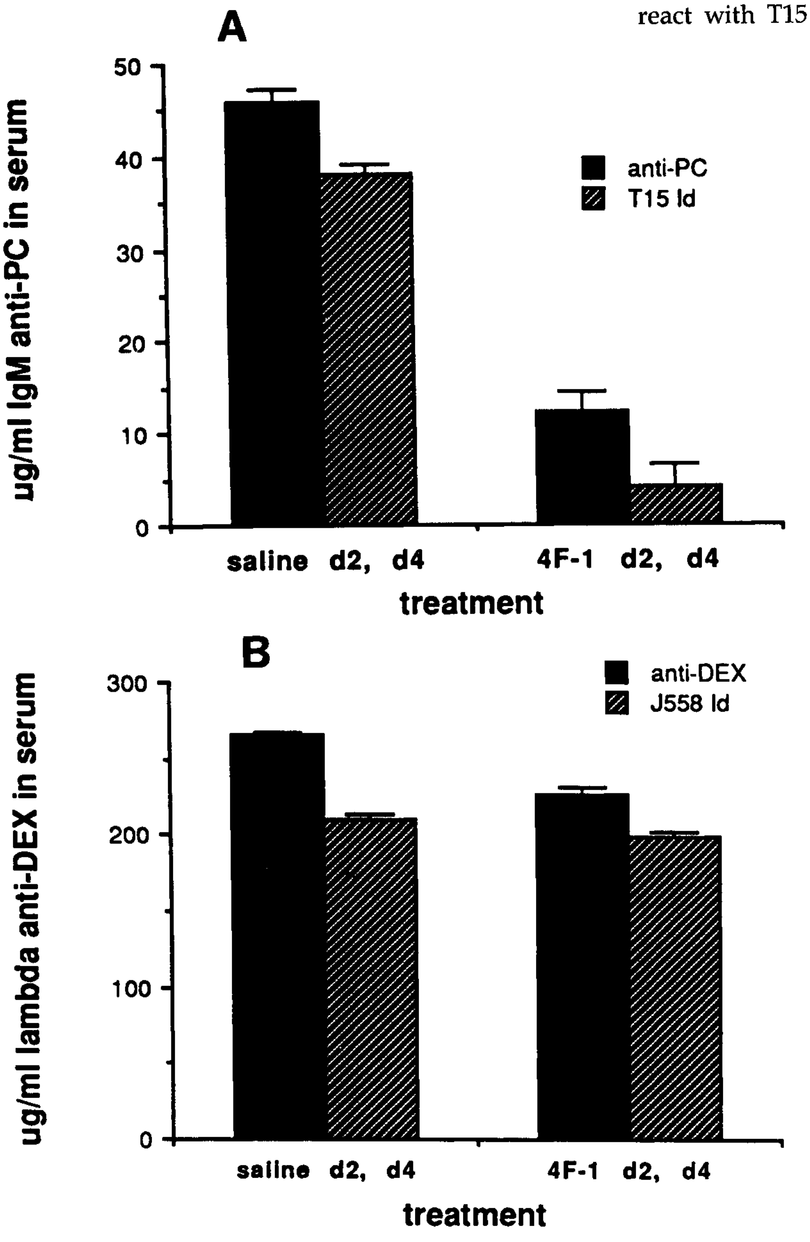

FIGURE 5. Effects of neonatal administration of the anti-M167 idiotype antibody 4F-1 on subsequent antibody responses to PC and DEX. (A) Levels of anti-PC antibodies (solid bars) and $T15^+$ antibodies (cross-hatched bars) in control mice and in mice treated with 4F-1 as indicated. (B) Levels of anti-DEX antibodies (solid bars) and $J558^+$ antibodies (cross-hatched bars) in control mice and in mice treated with 4F-1.

treatment failed to alter the antibody response to DEX (Fig. 5B). These observations lead us to conclude that the presence of functional B cells producing M167-like antibodies may assist in the development and expansion of B cells producing T15-like antibodies, but do not affect the development of J558-like B cells.

**Effects of Neonatal Exposure of Mice to PC and DEX**

The most striking feature of the observations described before was that soluble T15 and J558 could reciprocally inhibit the development of the J558 or T15 idiotypes *in vivo*. This added further support to our previous hypothesis that the development of T15- and J558-like B cells is regulated by a set of common autoantiidiotypic B cells. We previously demonstrated that exposure of 2–4-day-old mice to R36A resulted in the priming of T15 negative, largely M167-like antibodies, whereas exposure to the same antigen between days 6 and 21 primed for T15-like antibodies (Vakil et al., 1991). The next experiment was, therefore, designed to study connectivity between the T15 and J558 idiotypes in response to perinatal administration of PC and DEX antigens.

Groups of mice were injected with $2\times10^7$ R36A on days 2, 9, or 15 after birth and they were challenged with dextran at 7 weeks of age. As shown in Fig. 6A, in mice exposed to PC 2 days after birth, the antibody response to DEX when challenged as adults was greatly diminished, whereas in mice exposed to PC on day 9, a moderate response to DEX was observed although the J558 idiotype was diminished in proportion. In contrast, treatment of mice with PC at 15 days after birth did not affect the quantity or J558 portion of the anti-DEX response. In a similar experiment, mice were injected with 5 $\mu$g of DEX on days 2, 7, 14, or 21 after birth and challenged with R36A and DEX at 8 weeks of age (Fig. 6B). All DEX-treated mice mounted an enhanced response to DEX as compared to saline-treated controls. However, in mice treated with DEX on days 2 or 7 after birth, the J558 idiotype failed to dominate the immune response. All mice in this set also mounted normal anti-PC responses with dominant expression of the T15 idiotype (results not shown).

Thus, exposure to PC prior to the development of T15- and J558-like B cells resulted in a distortion of anti-DEX responses, however, exposure to DEX during these same windows of development had no effect on the anti-PC response. We have previously shown that the first DEX-specific precursors appearing in the neonatal period express the normally minor M104E idiotype and that J558-like B cells are not detected in the splenic focus assay until 12 to 15 days after birth (Stohrer and Kearney, 1984). This is reflected also by the priming effect of DEX treatment on days 2 and 7 (Fig. 6B), when these non-J558- anti-DEX precursors can be primed. Thus, although the injection of J558 antibodies into 2- or 8-day-old mice results in the modulation of T15 idiotype, B cells expressing the J558 idiotype may not be induced in mice before 12 to 15 days after birth, and, therefore, injection of dextran into mice before development of $T15^+$ B cells does not affect T15 expression later in life.

### DISCUSSION

Our previous studies indicated that B cells producing immunoglobulins that cross-react *in vitro* with purified antibodies expressing either the T15 or the J558 idiotype exist in neonatal mice and can be isolated in the form of hybridomas. The dual reactivity or cross-reactivity of one such autoantiidiotypic antibody BD2 has been confirmed by appropriate binding-inhibition assays (Vakil and Kearney, 1986). Administration of purified BD2 to neonatal mice was shown to enhance the development of both the T15 and the J558 idiotypes. As an extension of these previous findings, experiments were designed to address whether the idiotypic connectivity between T15 and J558 antibodies was functionally relevant with respect to the observed program of development of the B-cell repertoire. We, therefore, sought to test the idea that administration of soluble idiotype-bearing immunoglobulins would block signaling through B-cell-associated idiotypes on the precursors of endogenous T15- and J558-like B cells.

The first set of experiments demonstrated that administration of soluble antibodies expressing the T15 or the J558 idiotype specifically inhibits the development of both T15- and J558-idiotype-bearing B cells, supporting the existence of a common regulatory cell for both idiotypes. Next, we showed antibodies expressing the M167 idiotype that normally constitutes a minor component of PC-binding antibodies enhanced the response to PC while inhibiting the response to DEX. We have previously noted that antibody M167 enhances binding of BD2 to T15 *in vitro*, whereas it inhibits binding of the same antibody to J558 (Vakil and Kearney, 1986). This phenomenon may reflect changes in affinity of BD2 toward T15 and J558 resulting from conformational changes in the presence of M167 *in vitro*. Nevertheless, it prompted us to investigate the effects of HPCM27, a germline counterpart of M167, on endogenous T15- and J558-like B cells. The enhancement of anti-PC response with dominant expression of the T15 idiotype and elimination of the J558 idiotype following neonatal administration of HPCM27 provided perfect correlation between the *in vitro* properties and *in vivo* modulatory effects of antibodies bearing the M167 idiotype. These results suggest that although the mechanisms involved in the enhancement or suppression of T15 and J558 idiotypes by these neonatal antibody treatments are complex, the behavior of monoclonal antibodies *in vitro* correlates with these effects *in vivo* and can provide valuable clues to the nature of normal events in repertoire selection.

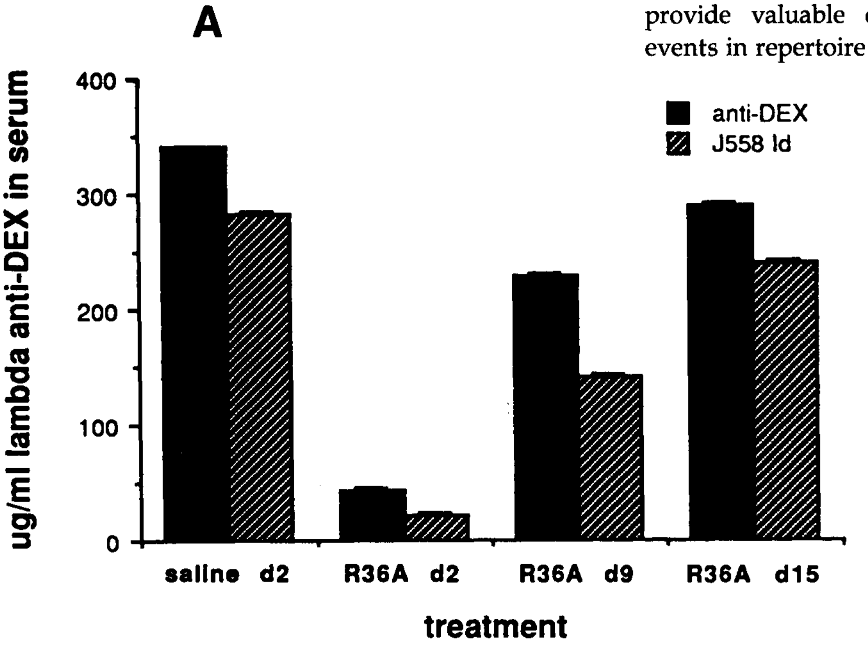

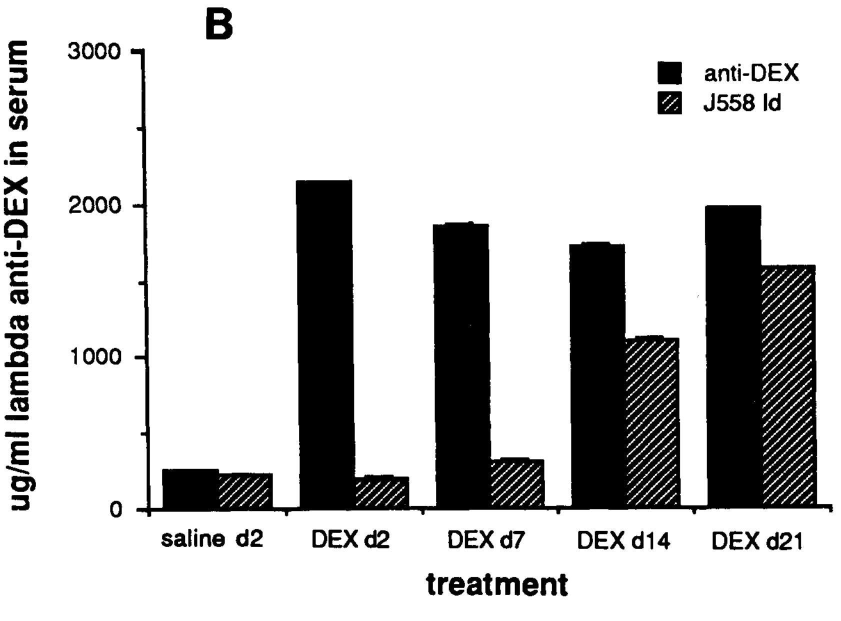

FIGURE 6. Effects of neonatal administration of R36A or dextran on the subsequent antibody responses to DEX. Levels of anti-DEX antibodies (solid bars) and J558[+] antibodies (cross-hatched bars) in control mice and in mice treated at ages indicated on the abscissa with (A) $2 \times 10^7$ R36A or (B) 5 $\mu$g dextran B1355S.

It is probable that M167-like B cells, which are amongst the earliest PC-responsive B cells to become functional (Vakil et al., 1991) may in fact serve as autoantiidiotypic B cells, as proposed in Fig. 1B, and aid in the idiotype-directed selection of T15-like B cells involving intermediate BD2-like B cells. The loss of T15 idiotype in mice treated with anti-M167 antibody 4F-1 supports this notion. The idiotype-directed signals generated by BD2-like B cells may induce a selective differentiation and/or expansion of the target T15-like B cells.

The reasons for inhibition of J558 idiotype by M167 are unclear. However, normal expansion of J558 idiotype occurs during the latter part of the second week of life in BALB/c mice. By this time, PC-specific B cells expressing the T15 idiotype are at adult levels. The initially predominant M167-idiotype-bearing B cells are now diluted and may not affect the development of the J558 idiotype. It is also possible that other anti-J558 B cells besides those represented by hybridoma BD2 are responsible for development of J558-like B cells during the second and third week of life in BALB/c mice.

One of the most striking findings in these studies was that exposure of newborn mice to R36A also resulted in the loss of the capacity to respond to DEX. This effect could also be mimicked by injection of antibodies expressing the M167 idiotype between days 4 and 12. These experiments added further evidence that functional idiotypic connections exist between sets of B cells specific for PC and DEX and that perturbation of the developing PC-responsive B cells can adversely affect development of those that bind to a structurally distinct antigen such as DEX, which appears later during development.

Based on our previous findings, the mininetwork described in Fig. 1 must represent only a small portion of the overall potential for interactions within the early B-cell repertoire. From the reactivity patterns of other hybridoma antibodies isolated from fetal and neonatal BALB/c mice, it is evident that the perinatal B-cell repertoire is highly connected through imnmunoglobulin idiotypes (Kearney et al., 1987). It is clear that during development, some clones increase in frequency while others decline (Sigal et al., 1977; Stohrer and Kearney, 1984; Vakil et al., 1991). The mechanisms involved in this waxing and waning of clones are not understood, however, they may involve competition for space, cytokines, or idiotype-directed signals. The fact that the pattern of development of the B-cell-specificity repertoire repeats itself generation after generation suggests that there is fine-tuning of these interactions among newly formed B cells in the neonate.

It is also possible that clones of B cells generated in the primary lymphoid organs during development may engage in idiotypic interactions prior to surface expression of conventional surface immunoglobulin. The expression of $V_H$ structures in conjunction with an invariant surrogate light-chain complex consisting of $\lambda 5$ and Vpre-B molecules would also provide a possibility for idiotypic selection to occur at the pre-B-cell level.

Our results show that interference with the developmental program of clonal selection can lead to serious deficiencies in the immune response to exogenous antigens later in life. We have recently obtained evidence that neonatal administration of PC also leads to a modulation of the immune response to group A Streptococcus (Vakil and Kearney, unpublished observations). It is likely that mice neonatally exposed to these antigens suffer from several other deficits in their B-cell repertoires that are as yet unknown to us. It is known that human infants do not respond to bacterial polysaccharide antigens until about 2 years of age and infections with *S. pneumoniae, H. influenzae,* and other Gram-positive bacteria are of common occurrence during infancy. The implications of our studies, therefore, go beyond the purpose, which is an attempt to understand the cellular events that lead to the selection and establishment of a normal adult B-cell repertoire in mammals. They emphasize the importance of vigorous immunological monitoring in human infants receiving synthetic conjugate vaccines containing bacterial polysaccharides that are currently being developed for the induction of immunity in human infants.

### METHODS

### Mice

Male and female BALB/c mice were obtained from Jackson laboratories, Bar Harbour, ME, and bred in our facility.

### Neonatal Treatments and Immunizations

Neonatal mice were injected with monoclonal antibodies in saline or $2 \times 10^7$ R36a or 5 $\mu$g dextran B1355S in saline by intraperitoneal route at ages indicated in the Results section. Control mice were injected with saline. Adult mice were immunized with $2 \times 10^8$ R36A in saline or dextran B1355S in saline intraperitoneally, and were bled 7 days later.

### Quantitation of Serum Antibodies

PC- and DEX-specific antibodies in sera of immunized mice were analyzed in a quantitative ELISA, as previously described (Stohrer and Kearney, 1984). Briefly, polyvinyl microtiter plates were coated with PC-BSA, DEX-BSA, or monoclonal antiidiotypic antibodies, blocked and incubated with serial dilutions of serum samples from individual mice, or known amounts of monoclonal standards. The assays were developed with phosphatase-labeled goat antimouse IgM antibodies and phosphatase substrate. The absorbance values were read in a Titertek multiscan (Flow Laboratories, McCleany, VA) and the standard equivalents were calculated based on reference O.D. values of monoclonal antibody standards. Data are represented as geometric means of groups of six to eight mice with standard deviations calculated using a rank test.

### ACKNOWLEDGMENTS

We wish to thank Ann Brookshire for her expertise in preparation of this manuscript. This work has been supported by NIH grants CA 13148, Al 30879, Al 14782, and Al 23694.

(*Received October* 16, 1990)

(*Accepted December* 10, 1990)

### REFERENCES

Behar S.M., Chien N.C., Corbet S., Diamond B., Getzoff E.D., Lustgarten D., Roberts V.A., Scharff M.D., and Chin S.-U. (1989). Impact of somatic mutation on the S107 (T15) heavy chain V region of antibodies reactive with self and non-self. *In:* Cold Spring Harbor Symposia on quantitative biology, vol. LIV. (Cold Spring Harbor, NY: Cold Spring Harbor Laboratory Press), pp. 921–931.

Bona A., Heber-Katz E., and Paul W.E. (1981). Idiotype-anti-idiotype regulation. I. Immunization with a levan-binding myeloma protein leads to the appearance of auto-anti- (anti-idiotype) antibodies and to the activation of silent clones. J. Exp. Med. **153:** 951–967.

Blomberg B., Geckeler W., and Weigert, M. (1972). Genetics of the antibody response to dextran in mice. Science **177:** 178–180.

Capra J.D., Slaughter C., Milner E.C.B., Estess P., and Tucker P.W. (1977). The cross-reactive idiotype of A-strain mice. Immunol. Today **3:** 332–339.

Cazenave P.-A. (1977). Idiotypic-anti-idiotypic regulation of antibody synthesis in rabbits. Proc. Natl. Acad. Sci. USA **74:** 5122–5125.

Cerny J., and Caulfield M.J. (1981). Stimulation of specific antibody-forming cells in antigen-primed nude mice by the adoptive transfer of syngeneic anti-idiotypic T cells. J. Immunol. **126:** 2262–2266.

Claflin J.L. (1976). Uniformity in the clonal repertoire for the immune response to phosphorylcholine in mice. Eur. J. Immunol. **6:** 669–674.

Claflin J.L., Lieberman R., and Davie J.M. (1974). Clonal nature of the immune response to phosphorylcholine. II. Idiotypic specificity and binding characteristics of anti-phosphorylcholine antibodies. J. Immunol. **112:** 1747–1756.

Cosenza H., and Köhler H. (1972). Specific inhibition of plaque formation to phosphorylcholine by antibody against antibody. Science **176:** 1027–1029.

Eichmann K. (1974). Idiotype suppression. I. Influence of the dose and of the effector functions of anti-idiotypic antibody on the production of an idiotype. Eur. J. Immunol. **4:** 296–302.

Gearhart P.J., Johnson N.D., Douglas R., and Hood L.E. (1981). IgG antibodies to phosphorylcholine exhibit more diversity than their IgM counterparts. Nature **291:** 29–34.

Hansberg D., Briles D.E., and Davie J.M. (1977). Analysis of the diversity of the murine response to dextran B1355. II. Demonstration of multiple idiotypes with variable expression in several strains. J. Immunol. **119:** 1406–1412.

Holmberg D., Forsgren S., Forni L., Ivars F., and Coutinho A. (1984). Reactions among IgM antibodies derived from normal neonatal mice. Eur. J. Immunol. **14:** 435–441.

Kang C.Y., and Köhler H. (1990). Characterization of an autologous monoclonal antibody against the MOPC167 idiotype. Korean Biochem. J. **23:** 296–301.

Kearney J.F., Vakil M., and Nicholson N. (1987). Non random $V_H$ gene expression and Idiotype anti-idiotype expression in early B cells. *In:* Evolution and Vertebrate Immunity: The antigen-receptor and MHC gene families, Kelsoe G., and Schulze D.H., Eds. Austin, TX: Texas University Press, pp. 175–190.

Kehry M., Sibley C., Fuhrman J., Schilling J., and Hood L.E. (1979). Amino acid sequence of a mouse immunoglobulin $\mu$ chain. Proc. Natl. Acad. Sci. USA **76:** 2932–2936.

Kelsoe G., and Cerney J. (1979). Reciprocal expansions of idiotypic and anti-idiotypic clones following antigen stimulation. Nature **279:** 333–334.

Lieberman R., Potter M., Humphrey W., Jr., and Chien C.C. (1976). Idiotypes of inulin-binding antibodies and myeloma proteins controlled by genes linked to allotype locus of the mouse. J. Immunol. **117:** 2105–2111.

Mäkelä O., and Karjalainen K. (1977). Inherited immunoglobulin idiotypes of the mouse. Immunol. Rev. **34:** 119–138.

Pawlak L., Hart D., and Nisonoff A. (1973). Requirements for prolonged suppression of an idiotype specificity in adult mice. J. Exp. Med. **137:** 1442–1458.

Pawlak L.L., and Nisonoff A. (1973). Distribution of a cross-reactive idiotypic specificity in inbred strains of mice. J. Exp. Med. **137:** 855–869.

Perlmutter R.M., Crews S.T., Douglas R., Sorensen G., Johnson N., Nivera N., Gearhart P.J., and Hood L. (1984). The generation of diversity in phosphorylcholine-binding antibodies. Adv. Immunol. **35:** 1–37.

Pollok B.A., and Kearney J.F. (1984). Identification and characterization of an apparent germline set of anti-idiotypic regulatory B lymphocyte. J. Immunol. **132:** 114–121.

Pollok B., Kearney J.F., Vakil M., and Perry R.P. (1984). A biological consequence of variation in the site of D-$J_H$ gene rearrangement. Nature **311:** 376–379.

Rajewsky K., and Takemori T. (1983). Genetics, expression, and function of idiotypes. Ann. Rev. Immunol. **1:** 569–607.

Rudikoff S., and Potter M. (1976). Size differences among immunoglobulin heavy chains from phosphorylcholine-binding proteins. Proc. Natl. Acad. Sci. USA **73:** 2109–2112.

Schilling J., Clevinger B., Davie J.M., and Hood L. (1980). Amino acid sequence of homogeneous antibodies to dextran and DNA

rearrangements in heavy chain V-region gene segments. Nature **283:** 35–40.

Sigal N.H., Pickard A.R., Metcalf E.S., Gearhart P.J., and Klinman N.R. (1977). Expression of phosphorylcholine-specific B cells during murine development. J. Exp. Med. **146:** 933–948.

Stohrer R., and Kearney J.F. (1984). Ontogeny of B cell precursors responding to $\alpha$1-dextran in BALB/c mice. J. Immunol. **133:** 2323–2326.

Vakil M., Briles D.E., and Kearney J.F. (1991). Antigen independent selection of T15 idiotype during B-cell ontogeny in mice. Dev. Immunol. **1:** 203–212.

Vakil M., and Kearney J.F. (1986). Functional characteristics of monoclonal atuo-anti-idiotype antibodies isolated from the early B cell repertoire of BALB/c mice. Eur. J. Immunol. **16:** 1151–1158.

Vakil M., Sauter H., Paige C., and Kearney J.F. (1986). *In vivo* suppression of perinatal multispecific B cells results in a distortion of the adult B cell repertoire. Eur. J. Immunol. **16:** 1159–1165.

